# The determinants associated with zoonotic potential of influenza A viruses: BTN3A3 evasion mediated by residue mutation in the nucleoprotein

**DOI:** 10.1002/mco2.441

**Published:** 2023-12-02

**Authors:** Lili Tian, Maochen Li, Huahao Fan

**Affiliations:** ^1^ College of Life Science and Technology Beijing University of Chemical Technology Beijing China

## Abstract

Mutation of residue 313 in the viral nucleoprotein from F/L to Y/V (or substitutions to N, H, or Q in the nucleoprotein residue 52 adjacent to residue 313) facilitates IAVs to escape from BTN3A3 restriction on virus replication.

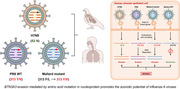

1

In a recently published study in *Nature* , Pinto et al.^1^ found that the residue 313 mutation in the viral nucleoprotein from F/L to Y/V (or substitutions to N, H, or Q in the nucleoprotein residue 52 adjacent to residue 313) facilitates avian influenza A viruses (IAVs) to escape from butyrophilin subfamily 3 member A3 (BTN3A3) restriction on virus replication.^1^ It is necessary to strengthen surveillance of the IAV variations associated with BTN3A3 evasion, which helps early risk assessment of the influenza pandemic occurrence and reduces the burden of global public health.^1^, ^2^

The zoonotic infections of IAVs cause severe diseases in humans, whose annual epidemics will result in approximately 3–5 million severe cases and about 290,000–650,000 respiratory deaths worldwide (https://www.who.int).^3^ It is of great significance to identify key host factors related to IAV infection and figure out how mutations in the viral genome facilitate cross‐species transmission.^4^ Influenza virus replication relies on amounts of molecular basis, while restrictive host factors, such as interferon‐stimulated genes (ISGs), play important roles in controlling or reducing infection.^4^ Arrayed expression screening of human (525) and macaque (345) ISGs on MT4 cells was performed,^1^ and above‐mentioned cells were infected with Mallard‐GFP (an H1N1 avian strain), PR8‐GFP (a human laboratory‐adapted H1N1 strain), and Cal04 7:1 PR8 segment 8 GFP (a mouse‐adapted H1N1 strain). A series of host antiviral factors were identified, including previously reported ISGs, such as IFITM2, IFITM3, and Mx1.^1^, ^2^, ^5^ Surprisingly, overexpression of BTN3A1 and BTN3A3 was found to restrict Mallard‐GFP rather than PR8‐GFP or maCal04‐GFP infection, implying that BTN3A3 specifically restricts avian IAVs.^1^ Further functional experiments validated that overexpression of BTN3A1 and BTN3A3 in A549 cells can effectively suppress H1N1 avian strain infection, while BTN3A3 but not BTN3A1 silencing promotes the infection of the Mallard‐IAV strain, indicating that BTN3A3 plays a more important role in restricting avian IAV replication than BTN3A1. The replication kinetics results also support above conclusion, and BTN3A3 overexpression can result in virus titer decrease of up to 100,000‐fold in A549 cells,> approximately 10,000 times higher than that of BTN3A1‐overexpressing cells. Different from Mallard‐IAV's sensitivity to BTN3A1 and BTN3A3, PR8 can evade either human BTN3A protein, which suggests that sequence differences between PR8 and Mallard‐IAV genomes may participate in BTN3A3 evasion.^1^

BTN3A proteins are primate‐specific paralogs, and proteins homologous to the human BTN genes expressed by other species cannot restrict PR8 or Mallard‐IAV infection.^1^, ^5^ Most BTN3A1 and BTN3A3 genes consist of an N‐terminal IgV, IgC, and a PRYSPRY domain, which is devoid in BTN3A2, explaining its incapability in restricting IAV infection (Figure 1A).^1^ Further studies demonstrated that BTN3A3 inhibits avian IAV RNA replication in the early stages of the virus lifecycle.^1^ Moreover, the restrictive effect is specific to inhibiting avian IAVs rather than other respiratory viruses, such as severe acute respiratory syndrome coronavirus 2 and respiratory syncytial virus.^1^ In particular, BTN3A3 is not the sole species barrier of zoonotic infections with IAVs.^1^ As another molecular basis preventing spillover transmission of avian IAVs, Mx1 encodes myxovirus resistance protein A (MxA), which recognizes and blocks viral nucleoprotein from entering into the nucleus of infected cells.^5^ The antiviral mechanism and sites of action are similar between BTN3A3 and Mx1, and their antiviral activity is exerted by inhibiting the transcription and replication of the viral genome at an early stage.^2^, ^5^ However, both in vitro and in vivo data indicated that BTN3A3 and Mx1 exert their restrictive effects on IAV replication independently.^1^, ^2^

**FIGURE 1:**
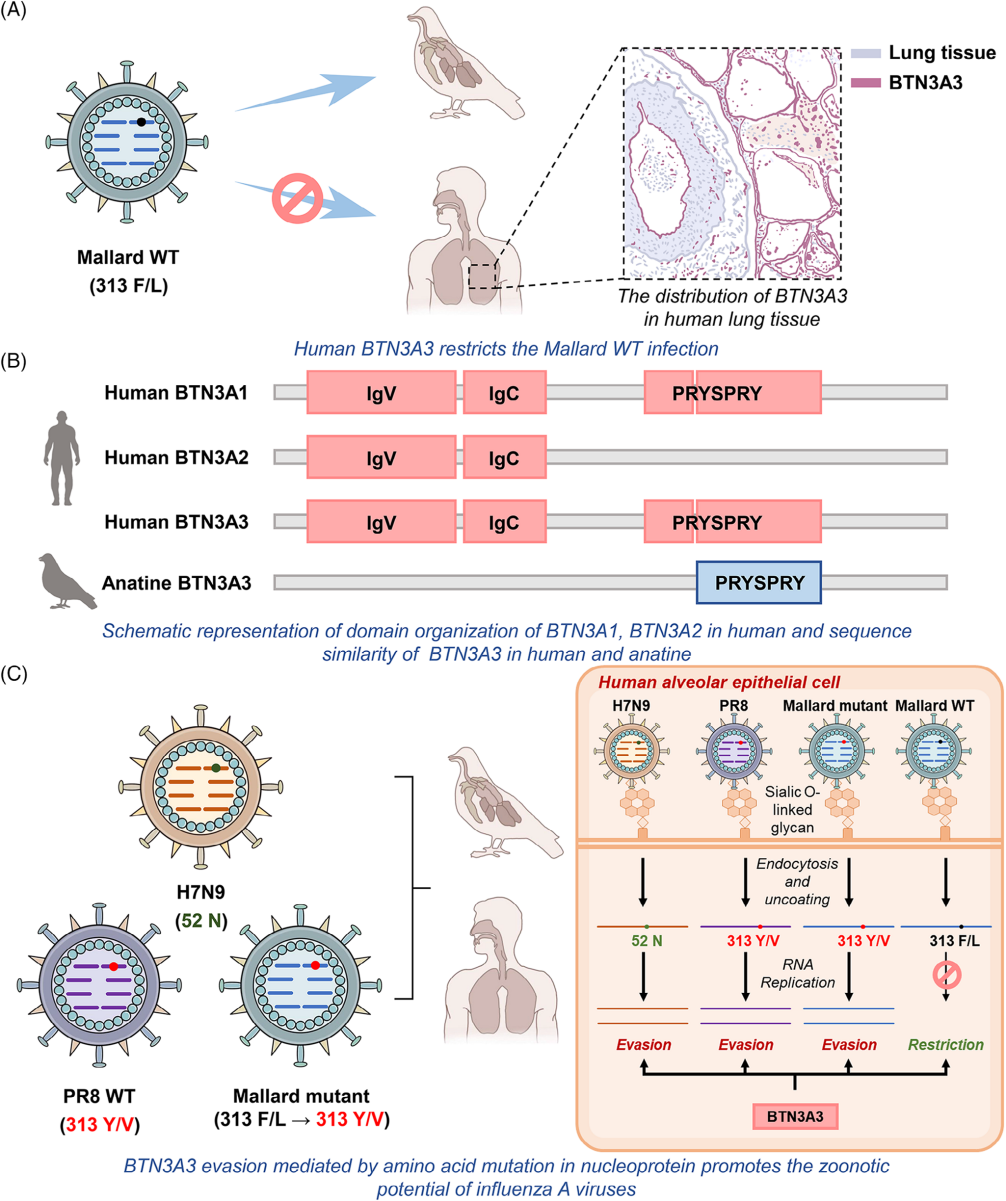
Butyrophilin subfamily 3 member A3 (BTN3A3) evasion mediated by amino acid mutation in the nucleoprotein promotes the zoonotic potential of influenza A viruses (IAVs). (A) Mallard‐IAV wild type (WT) (313F/L in the nucleoprotein) can infect avian but not human due to BTN3A3 restriction, and BTN3A3 is constitutively expressed in the respiratory tract of healthy human but not avian. (B) Schematic representation of domain organization and sequence similarity of BTN3A in human and anatine. The dissimilarities between two domains explain the incapability in restricting IAV infection. (C) The H7N9 (313F+52N in the nucleoprotein), PR8 WT (313Y/V in the nucleoprotein), and Mallard‐IAV variant (313Y/V in the nucleoprotein) can evade BTN3A3 restriction and infect both human and avian. Conversely, Mallard‐IAV with 313F/L in the nucleoprotein can only infect avian.

IAVs are enveloped negative‐sense single‐strand RNA viruses with eight RNA genomic segments, encoding RNA polymerase subunits, viral glycoproteins (hemagglutinin and neuraminidase), viral nucleoprotein, matrix protein (M1) and membrane protein (M2), the nonstructural protein NS1, and nuclear export protein.^3^, ^5^ To ascertain determining factors for the sensitivity to BTN3A3 in avian IAVs, the PR8/Mallard‐IAV recombinant viruses with different fragments (7:1) were constructed and the plaque forming assay was used to measure the infectivity in Madin–Darby canine kidney (MDCK) cells overexpressing BTN3A3. Replacement of fragment 5 (nucleoprotein) in avian H1N1 with corresponding sequences of PR8 endowed the ability to escape from BTN3A3 restriction. The influenza viral nucleoprotein plays a crucial role in viral RNA replication.^4^ Comparison of the nucleoprotein sequences between Mallard‐IAV and PR8 identified several amino acid mutations including V33I, R100V, L136M, F313Y, R351K, V353L, and Q357K from Mallard‐IAV to PR8.^1^ According to the loss‐of‐function experiment, F313Y was found to be the key mutation site in the avian H1N1 IAV that mediates BTN3A3 evasion (Figure 1B).^1^

Subsequently, phylogenetic tree analysis of over 30,000 IAV nucleoprotein amino acid sequences showed that almost all IAVs causing human infection had a nucleoprotein with a 313 residue of Y or V.^1^ IAVs containing the nucleoprotein with 313 V residues can infect human, swine, and avian, which could be traced back to the 1918 H1N1 pandemic and cause many seasonal epidemics.^1^, ^2^ Moreover, IAVs with 313Y residues can only infect human and avian. Correspondingly, F313V mutation occurred in IAV‐infected swine during 2002−2006 (Figure 1C).^3^

Surprisingly, residue 313 of the nucleoprotein in avian and human isolates of H7N9 is F,^2^ which might be attributed to the presence of 52Y in the nucleoprotein facilitating to evade BTN3A3 restriction. Additionally, the 52 residue mutation of Q or H in either avian H1N1 (313F) or H7N9 (313F) backbone can also evade BTN3A3 restriction (Figure 1C).^1^, ^2^ Both residues 313 and 52 are situated on the surface of the nucleoprotein trimer and tightly arranged within the nucleoprotein head domain, implying that these two residues can interact with other viruses and host factors.^1^, ^5^

Immunohistochemistry found that the upper and lower respiratory tracts of healthy human donors both express BTN3A3 constitutively (Figure 1B), and type I or II IFNs can significantly upregulate BTN3A3 expression. Compared with the control group, overexpression of BTN3A3 potently protected C57BL/6 mice from pulmonary infection by Cal04 mutant (313F) but not Cal04 wild type (313 V).^1^

The spillover of avian IAVs from avian to human has caused several global pandemics, seriously affecting global economic development and threatening the health of humans and animals.^3^, ^4^ The H1N1 pandemic in 1918 and H2N2 pandemic in 1957 might be all related to BTN3A3 evasion mediated by mutations in residue 313 or related residues in the nucleoprotein of IAVs. Recently, increasing fatal outbreaks caused by IVAs (H5) in mammals might serve as a recombination vector for genome segment exchange of influenza viruses, leading to the emergence of new highly pathogenic viruses in animals and humans (https://www.who.int). It is worrisome that regional migration of migratory birds, intensified livestock production, and global population mobility accelerate the cross‐species transmission of IAVs from other host species to humans.^3^ Close surveillance and monitoring of viral adaptive mutations related to BTN3A3 and Mx1 evasion could be considered in the further studies.^1^, ^2^ Moreover, timely and effective warning and prevention measures should be taken to reduce the impact of related viral mutations on human production and life.

## AUTHOR CONTRIBUTIONS

H.F. designed the research. H.F., L.T., and M.L. read the papers and analyzed the data. L.T. and H.F. wrote and revised the manuscript. All authors have read and approved the final manuscript.

## CONFLICT OF INTEREST STATEMENT

The authors declare they have no conflicts of interest.

2

## ETHICS STATEMENT

Not applicable.

## Data Availability

Not applicable.
